# Novel Insights into the Molecular Regulation of Ribonucleotide Reductase in Adrenocortical Carcinoma Treatment

**DOI:** 10.3390/cancers13164200

**Published:** 2021-08-20

**Authors:** Christina Bothou, Ashish Sharma, Adrian Oo, Baek Kim, Pal Perge, Peter Igaz, Cristina L. Ronchi, Igor Shapiro, Constanze Hantel

**Affiliations:** 1 Department of Endocrinology, Diabetology and Clinical Nutrition, University Hospital Zurich (USZ), University of Zurich (UZH), CH-8091 Zurich, Switzerland; Christina.Bothou@usz.ch (C.B.); converse.ashish@gmail.com (A.S.); Igor.Shapiro@usz.ch (I.S.); 2 Competence Centre of Personalized Medicine, Molecular and Translational Biomedicine PhD Program, University of Zurich (UZH), CH-8006 Zurich, Switzerland; 3 Department of Pediatrics, School of Medicine, Emory University, Atlanta, GA 30322, USA; adrian.oo@emory.edu (A.O.); baek.kim@emory.edu (B.K.); 4 Center for Drug Discovery, Children’s Healthcare of Atlanta, Atlanta, GA 30322, USA; 5 Department of Endocrinology, Department of Internal Medicine and Oncology, Faculty of Medicine, Semmelweis University, H-1083 Budapest, Hungary; paul.perge@gmail.com (P.P.); igaz.peter@med.semmelweis-univ.hu (P.I.); 6 MTA-SE Molecular Medicine Research Group, H-1083 Budapest, Hungary; 7 Division of Endocrinology and Diabetes, Department of Medicine I, University Hospital of Wuerzburg, University of Wuerzburg, 97080 Wuerzburg, Germany; C.L.Ronchi@bham.ac.uk; 8 Institute of Metabolism and Systems Research, University of Birmingham, Birmingham B15 2TT, UK; 9 Centre for Endocrinology, Diabetes and Metabolism, Birmingham Health Partners, Birmingham B15 2TT, UK; 10 Medizinische Klinik und Poliklinik III, University Hospital Carl Gustav Carus Dresden, 01307 Dresden, Germany

**Keywords:** adrenocortical carcinoma, adrenocortical cell line, RRM2, RNR, COH29

## Abstract

**Simple Summary:**

The current clinical gold standard etoposide, doxorubicin, cisplatin, and mitotane (EDP-M) is not satisfying for the treatment of adrenocortical carcinoma (ACC). However, clinical translation of novel, preclinically promising therapies were unfortunately disappointing in recent years, indicating that utilized tumor models inadequately predicted clinical applicability of novel pharmacological approaches. In an attempt to optimize the current preclinical armamentarium, our workgroup initiated a comparative drug screen of clinically relevant chemotherapies and therapies targeting IGF, EGF, and Wnt signaling pathways in the classical NCI-H295R cell line and, for the first time, in the recently developed highly drug-resistant MUC-1 cell line. These testings revealed gemcitabine and cisplatin as a promising combination, but further investigations also indicated developing drug resistance mechanisms on the molecular level. We aimed to decipher underlying resistance mechanisms, identified ribonucleotide reductase as an important player, and successfully targeted the involved DNA damage/repair mechanism.

**Abstract:**

Current systemic treatment options for patients with adrenocortical carcinomas (ACCs) are far from being satisfactory. DNA damage/repair mechanisms, which involve, e.g., ataxia-telangiectasia-mutated (ATM) and ataxia-telangiectasia/Rad3-related (ATR) protein signaling or ribonucleotide reductase subunits M1/M2 (RRM1/RRM2)-encoded ribonucleotide reductase (RNR) activation, commonly contribute to drug resistance. Moreover, the regulation of RRM2b, the p53-induced alternative to RRM2, is of unclear importance for ACC. Upon extensive drug screening, including a large panel of chemotherapies and molecular targeted inhibitors, we provide strong evidence for the anti-tumoral efficacy of combined gemcitabine (G) and cisplatin (C) treatment against the adrenocortical cell lines NCI-H295R and MUC-1. However, accompanying induction of RRM1, RRM2, and RRM2b expression also indicated developing G resistance, a frequent side effect in clinical patient care. Interestingly, this effect was partially reversed upon addition of C. We confirmed our findings for RRM2 protein, RNR-dependent dATP levels, and modulations of related ATM/ATR signaling. Finally, we screened for complementing inhibitors of the DNA damage/repair system targeting RNR, Wee1, CHK1/2, ATR, and ATM. Notably, the combination of G, C, and the dual RRM1/RRM2 inhibitor COH29 resulted in previously unreached total cell killing. In summary, we provide evidence that RNR-modulating therapies might represent a new therapeutic option for ACC.

## 1. Introduction

Adrenocortical carcinoma (ACC) is a rare and highly metastatic malignancy with an estimated incidence of 0.7–1 cases per million population per year [1]. Initial diagnosis of ACC patients could be asymptomatic or can present with symptoms of a large, locally invasive primary tumor. Development of novel treatment modalities is impeded by heterogeneity among patients [2,3]. Currently, the only curative option for localized ACC tumors includes complete tumor resection, followed by adjuvant treatment with mitotane [1]. A combination of etoposide–cisplatin–doxorubicin (EDP-M) with mitotane constitutes the current gold standard for metastatic ACC, which, however, is not satisfactory and, furthermore, often results in clinical toxicity [1]. Overall survival and response rates for ACC are still disappointing, with an average survival from diagnosis of 15 months and a 5-year survival of around 20% [2,4]. Up to now, different molecular targeted therapies have been tested against ACC, including treatments targeting epidermal growth factor receptor (EGFR), vascular endothelial growth factor (VEGF), multityrosine kinases, or the insulin-like growth factor 1 receptor (IGF-1R) inhibitor. However, these treatments did not result in better overall survival or progression-free rates vs. placebo [5,6].

Ribonucleotide reductase (RNR) could be a potentially promising therapeutic target in this context. RNR is the only enzyme that catalyzes de novo formation of deoxyribonucleotide triphosphates (dNTPs). Thus, it is a key enzyme in DNA synthesis and repair [7]. RNR is a tetramer consisting of two homodimeric subunits, the large catalytic dimer ribonucleotide reductase subunit M1 (RRM1), encoded by *RRM1* gene, and the small regulatory dimers RRM2 or p53R2, encoded each by distinct genes, *RRM2* and *RRM2B*, respectively. RRM1/RRM2 holoenzyme contributes to S-phase nuclear DNA replication and repair in proliferating cells, while RRM1/p53R2 provides dNTPs for nuclear DNA repair [7]. Elevated levels of both RRM1 and RRM2 subunits occur in various human cancers, making RNR a potential therapeutic target [8]. Moreover, there is increasing evidence that RRM1 and RRM2 accumulate at DNA damage sites in the nucleus when appropriate damage is induced by chemotherapy [8]. Thus, enhanced expression of *RRM1, RRM2*, or *RRM2B* may be upregulated to increase abundance and activity of RNR to supply affected cells strongly with essential dNTPs. Accordingly, improved disease-free survival upon adjuvant mitotane treatment was previously reported for ACC patients under low *RMM1* levels exclusively [9]. Moreover, comparative reanalysis of publicly available microarray datasets indicated upregulation of *RRM2* in ACC compared to adrenocortical adenoma (ACA) and that, in selected ACC samples, *RRM2* correlated well with the specific Ki67 indices of these tumors [10]. Additionally, *RRM2* upregulation has been involved in resistance to gemcitabine in clinical series of different neoplasms [8]. In accordance, *RRM2* was furthermore found to be upregulated upon G treatment in the adrenocortical cell line NCI-H295R. However, the subsequent aim to decrease its expression in combination with other drugs failed, as these drugs did not alter G effect [10].

Here we report for the first time on a therapeutic combination of gemcitabine (G), cisplatin (C), and the dual RNR-inhibitor COH29, which is able to strongly modulate the expression of genes coding for the RNR subunits, RNR protein abundance, RNR activity, and overall cell viability in preclinical models for ACC.

## 2. Materials and Methods

### 2.1. Cancer Cell Lines

NCI-H295R cells were originally obtained from ATCC and again authenticated by Microsynth (Balgach, Switzerland) in December 2020. MUC-1 cells were previously established by our group and again authenticated in December 2020 [11]. Both cell lines were maintained as previously described [11].

### 2.2. Si-RNA Experiments

When a confluence of 60–80% was reached, NCI-H295R (200,000/well) and MUC-1 cells (85,000/well) were seeded in 24-well plates (TPP #92024, Trasadingen, Switzerland) in penicillin/streptomycin-free medium and incubated for 24 h. For small interfering RNA (siRNA)-mediated knockdown of RRM2, cells were transfected with 100 nM of either the targeting or control (siRNA SMARTpool: ON-TARGETplus RRM2 siRNA #L-010379-00-0005 and ON-TARGETplus Non-targeting Pool #D-001810-10-05, Dharmacon, Lafayette, CO, USA) using Lipofectamine RNAiMAX Reagent (#3778030, Invitrogen^TM^, Waltham, MA, USA) in Opti-MEM medium (#31985062 Gibco^TM^, Waltham, MA, USA) for 24, 48, and 72 h. Then, the samples were collected for qPCR and Western blot using the respective buffers.

### 2.3. MTT Viability Assay

For cell viability assay (MTT), NCI-H295R (8000/well) and MUC-1 cells (6000/well) were seeded on a 96-well plate and incubated for 24 h. Drug-, inhibitor-, or combination-treated cells and their respective blanks and controls were incubated for 24 h, if not stated otherwise. For quantification, MTT dye at a final concentration of 0.5 mg/mL (#M5655, Sigma-Aldrich, Buchs, Switzerland) was added to the cells and incubated for 2 h (37 °C, 5% CO_2_), and then solubilized by acidified SDS solution (10% SDS/0.01 M HCl). After overnight incubation on an orbital plate shaker at room temperature in the dark, the plate was centrifuged and the absorbance was measured at 570 nm (reference wavelength: 650 nm) in a cell-imaging multi-mode reader (Cytation 5, BioTek Instruments, Winooski, VT, USA). For each well, the normalized cell viability was calculated as percentage of untreated with the following formula:

Viability (% of untreated) = ((delta 570–650 nm minus the respective delta of the blank wells of each treatment) mean of untreated) × 100%.

For the MTT assay, the respective cell-free but equally treated wavelength average for each control triplicate has been used as a blank.

For this study, all samples were analyzed on one day in one analytical run.

### 2.4. BrdU Proliferation Assay

BrdU assay was performed using cell proliferation ELISA (#11647229001, BrdU, colorimetric immunoassay) (Roche, Basel, Switzerland) for quantification following the manufacturer’s instructions. The same number of cells, and cell incubation for 24 h after cell seeding, as in the MTT assay was followed. After 24 h, the different treatments were added and the cells were incubated for different time points, as indicated in the description of each experiment. For the quantification, the absorbance was recorded at 450 nm (reference wavelength: 690 nm) without the lid in the cell-imaging multi-mode reader (Cytation 5). For data analysis, the normalized proliferative activity in each well was calculated as percentage of untreated with the following formula:

Proliferative activity (% of untreated) = ((delta 450–690 nm)/mean of untreated) × 100%.

### 2.5. Clonogenic Assay

When confluence of 60–80% was reached, NCI-H295R (30,000/well) and MUC-1 cells (20,000/well) were seeded in 6-well plates (TPP #92006) and incubated for 24 h. The medium was replaced with medium containing the treatment in the concentration stated in the different experiments, and the respective controls, and incubated on day 7. The plates were incubated until day 28. Following this, the media was removed and, after washing the plates with PBS (#10010023, Gibco^TM^), the plates were incubated with methanol and acetic acid solution for 30 min. After the aspiration of the fixative solution, the plates were stained with crystal violet solution (0.5% *w*/*v*) (#US029-500, Artechemis AG, Zofingen, Switzerland). The plates were dried overnight and the clones were counted on the last day using a stereomicroscope (Axiovert 25/HBO50, Zeiss, Oberkochen, Germany). Clonogenic cell survival was determined by the ability of single cells to form colonies in vitro, as previously described [12].

### 2.6. dATP Measurements

For each cell line, 2 million cells were seeded in a 60 mm dish and incubated for 24 h. The cell monolayer was washed twice with PBS and lysed by quickly adding ice-cold 65% methanol (100 μL per 1 × 10^6^ cells). The cells were scraped off the plate and another 100 μL of ice-cold 65% methanol were used for another washing of the plate and for the volume recovery. The samples were vortexed for 2 min and the cells were completely lysed by incubation at 95 °C for 3 min. After chilling of the tubes on ice for 1 min, the samples were centrifuged for 3 min at 14 K RPM. The supernatant was transferred to a new labeled tube with identifiable numbers, and the samples were dried by speed vacuum. The dried pellets were subsequently resuspended in dNTP buffer (50 mM Tris-HCl, pH 8.0 and 10 mM MgCl2) and approximately 1~2 μL of the extracted dNTP samples were used for each 20 μL single nucleotide incorporation reaction. The proper dilutions of the dNTP samples were prepared for the assay in order to make the primer extension values lie within the linear ranges of the dNTP incorporation (2~32% primer extension). The extracted dNTP samples were stored at −70 until used. Several different volumes of the extracted dNTP samples were also used to confirm the linearity of the primer extension. In addition, the dNTP samples were prepared from different cell numbers, depending on the recovery efficiency. The dNTP content of each cell type was quantified and normalized by pmole/1 × 10^6^, as per a previously published protocol [13].

### 2.7. Quantitative Real-Time PCR

For these experiments, NCI-295R (200,000/well) and MUC-1 cells (85,000/well) were seeded in 24-well plates (TPP #92024) and the drug treatment took place as described below. At certain time points, cells were washed with cold PBS twice and RNA lysis buffer was added on the top of the cells. Cells were scraped off into separate tubes and total RNA isolation followed, according to the manufacturer’s guidelines (#74104, RNAeasy kit, Qiagen, Hilden, Germany). Genomic DNA was removed after treatment with a Turbo-DNA free kit (#AM1907, ThermoScientific^TM^, Waltham, MA, USA). In total, 300–500 ng of RNA was transcribed into cDNA (#K1631, RevertAid™ H Minus First Strand cDNA Synthesis Kit with Random Hexamer Primers, ThermoScientific^TM^) according to the protocol of the producer. For real-time PCR analysis, we utilized the EvaGreen^®^ reaction mix (#1725200, Bio-Rad, Hercules, CA, USA) in the 7500 Fast Real-Time PCR Cycler (ThermoScientific^TM^). The primers used were human *RRM2* forward: CTGGCTCAAGAAACGAGGACTG; human *RRM2* reverse: CTCTCCTCCGATGGTTTGTGTAC (#HP203086 premixed, Origene, Rockville, MD, USA); human *RRM2b* forward: ACTTCATCTCTCACATCTTAGCCT; human *RRM2b* reverse: AAACAGCGAGCCTCTGGAACCT (#HP211794 premixed, Origene); human *RRM1* premixed, purchased from Realtimeprimers (#VHPS-8020, Elkins Park, PA, USA). Quantification was adjusted using the housekeeping gene *GAPDH* for human samples forward: 5′-AGC-CTC-CCG-CTT-CGC-TCT-CT-3′ and reverse: 5′-CCA-GGC-GCC-CAA-TAC-GAC-CA-3′ (Microsynth). Reactions were performed under the following conditions: 1 cycle at 95 °C for 3 min, 40 cycles at 95 °C for 5 s, 60 °C for 30 s. Differences of the threshold cycle (Ct) values between the GAPDH housekeeping gene and the gene of interest (ΔCt) were then calculated as an indicator of difference in the amount of mRNA expressed, corrected for the efficiency of the reaction previously acquired via standard curve.

### 2.8. Immunofluorescence

NCI-H295R (100,000 cells/well) and MUC-1 (80,000 cells/well) were seeded in 4-well chamber slides (#94.6190.402, Sarstedt, Nümbrecht, Germany) and incubated overnight (25 µM gemcitabine or 25 µM gemcitabine, and 40 µM cisplatin). Afterwards, cells were washed twice with PBS and fixed with 1 mL/well of freshly prepared 0.04 *v*/*v* paraformaldehyde solution for 30 min at room temperature. Following two washing steps with PBS, immunofluorescence staining for RRM2 and RRM1 was conducted according to the protocol for immunohistochemistry. Primary anti-RRM2 mouse MAB was used (#ab57653, Abcam, Cambridge, UK) at a 2.5 mg/mL dilution overnight at 4 °C. The secondary antibody was used at a 1:1000 dilution (GE Healthcare) for 1 h at room temperature. RRM2 immunostaining was evaluated by confocal microscopy using an Axoiplan 2 (Zeiss, Oberkochen, Germany), and images were scanned at 20× and 40× magnifications. All assays were repeated as independent experiments at least thrice.

### 2.9. Western Blotting

For Western blotting experiments, NCI-H295R (1,000,000/well) and MUC-1 cells (425,000/well) were seeded in 6-well plates (TPP#92006) and the drug treatment took place as described below. Cell proteins were extracted in RIPA buffer (50 mM Tris pH8.0, 150 mM NaCl, 0.01 *v*/*v* NP-40 #74385, Sigma-Aldrich, St. Louis, MI, USA), 0.005 *v*/*v* sodium deoxycholate (#D6750, Sigma-Aldrich), and 0.001 *w*/*v* SDS (#2326.3, Roth, Karlsruhe, Germany) supplemented with complete protease inhibitor cocktail (#11836170001, Roche, NY, USA) and phosphatase inhibitor cocktail (#P5726, Sigma-Aldrich).

The homogenized lysate was centrifuged at 16,000 g for 15 min and protein concentration was quantified by Pierce BCA Protein Assay (#23225, ThermoScientific^TM^) following the manufacturer’s recommendations. A total of 50 micrograms of protein solution was resuspended in Laemmli 2× loading buffer (4% SDS, 10% 2-mercaptoethanol, 20% glycerol, 0.004% bromophenol blue, and 0.125 M Tris-HCl) and boiled for 5 min at 95°C. Sample separation was carried out in a 0.1 *v*/*v* polyacrylamide gel and then transferred onto a PVDF membrane. After blocking in 0.05 *v*/*v* non-fat milk (diluted in TBST (137 mM NaCl, 2.7 nM KCl (Sigma-Aldrich), 19 mM TrisHCl, 0.1% Tween^®^20 (#85113, Sigma-Aldrich))) at room temperature for 1 h, membranes were incubated overnight with primary antibodies, following three washes for 5 min at RT and incubation for 1 h at RT with corresponding secondary antibodies diluted in 0.05 *v*/*v* non-fat dry milk. After three additional washes, proteins of interest were detected using Super Signal West Pico Plus (2 mL/membrane) (#34580, ThermoScientific^TM^) in a Molecular Imager^®^ ChemiDoc™ XRS+ with Image Lab™ Software (Bio-Rad).

Antibodies used: RRM2 (#ab57653, Abcam), rabbit Ser 345 p-Chk1 (#2348, Cell Signaling, Danvers, MA, USA), rabbit Thr68 p-Chk2 (#2661, Cell Signaling), p-H2AX (#9718, Cell Signaling), and beta- actin (#A5316, Sigma-Aldrich). Secondary antibodies: anti-rabbit HRP-linked (#NA9340V, GE Healthcare, Chicago, IL, USA) and anti-mouse HRP-linked (#NA931V, GE Healthcare). The Western blot quantification was done using densitometric analysis by ImageJ (Bethesda, MA, USA, https://imagej.nih.gov/ij/, (version 1.52a, accessed on 23 April 2018)). The original Western blot image in JPEG format was opened in ImageJ and quantification was done for both the protein of interest and loading control to obtain normalized pixel density. All assays were repeated as independent experiments at least thrice. The original Western blot images can be found at Figures S1–S4.

### 2.10. Drug Treatment Assays

Cells were seeded on different plates as described above for the different experiments. For the concentration–response curves, cells were exposed to increasing concentrations of chemotherapeutic agents and inhibitors. On the treatment starting day (24 h after cell seeding), the drug treatments were prepared in reaction tubes, as well as their respective controls. The cell medium was removed, the wells were washed with PBS, and the treatment agents were added on the top of the cells. Each concentration of the samples and each control was included in triplicate. The plate was incubated at 37 °C and 5% CO_2_ for different time points, and then the plates were processed accordingly. The chemotherapeutic and cytostatic agents that were used are as follows: cisplatin (#20J17LA, Teva, Basel, Switzerland), gemcitabine (#0B55015, Sandoz, Rotkreuz, Switzerland), etoposide (#HP0270, Sandoz), doxorubicin (#HK4929, Sandoz), and paclitaxel (#7J04629, Bristol-Myers Squibb SA, Steinhausen, Switzerland), all diluted in medium. Moreover, we used erlotinib (#10483, Cayman, Ann-Arbor, MI, USA), linsitinib (#17708, Cayman), sorafenib (#10009644, Cayman), XAV-939 (#S1180, Selleckchem, Houston, TX, USA), isoquercitrin (#S3842, Selleckchem), and sunitinib (#13159, Cayman), all in DMSO solvent, as well as 9-cis retinoic acid (#R4643, Sigma-Aldrich) and mitotane (#SML1885, Sigma-Aldrich) prepared in ethanol. The ready-to-use inhibitors that were used, VE822 (#S7102, Selleckchem), COH29 (#HY-19931), adavosertib (#HY-10993), prexasertib (#HY-18174), and AZD0156 (#HY-100016) (all by MedChem Express, Junction, NJ, USA), were diluted directly in medium.

### 2.11. Adrenal Tissue Samples and Clinical Characteristics

We also investigated the gene expression levels of *RRM1* and *RRM2* in two cohorts of fresh-frozen human adrenal tissues, including normal adrenal glands (NAG), adrenocortical adenomas (ACA), and ACC, i.e., a dataset available from a published microarrays study [14] and our own series. Our cohort included a total of 21 NAGs (18 derived from the area surrounding the tumors and 3 from adrenalectomies performed during surgery for renal carcinoma), 20 ACAs, and 30 ACCs. Clinical parameters, such as tumor size, hormone secretion pattern, and, in the case of ACC, tumor stage according to the European Network for the Study of Adrenal Tumors (ENSAT) classification [1,15], Weiss score, Ki67 proliferation index, and clinical outcome were collected. Demographic and clinical data are reported in Table 1.

### 2.12. RRM1 and RRM2 Expression in Adrenal Tissue Samples

In our cohort, the mRNA expression of *RRM1* and *RRM2* were evaluated by quantitative real-time PCR (qRT-PCR). In brief, RNA was isolated from fresh-frozen tissue samples using the RNeasy Lipid Tissue Minikit (Qiagen, Hilden, Germany). Reverse transcription of 1 μg of RNA was performed using the QuantiTect Reverse Transcription Kit (Qiagen) according to the manufacturer’s recommendations. Predesigned Taqman^®^ gene expression assays for *RRM1* (Hs01040698_m1) and RRM2 (Hs00357247_g1) (Applied Biosystems, Darmstadt, Germany) were used. *Beta actin* (Hs9999903_m1) expression was used for normalization. Remaining conditions for qRT-PCR were applied as previously published [16]. Transcript levels were determined using Bio-Rad CFX Manager 2.0 software and normalized to those of the housekeeping gene using the ΔCT method (Pfaffl Method), as previously described [16].

### 2.13. Statistical Analysis

**Figure 1:**
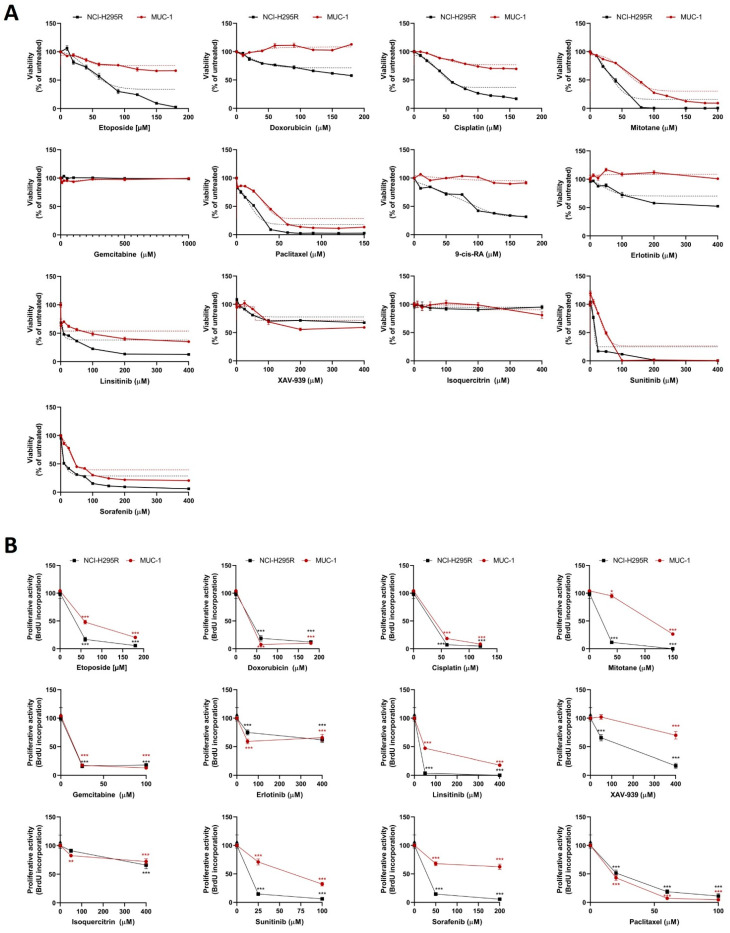
MTT viability assay of NCI-H295R and MUC-1 cells for different chemotherapeutic agents incubated for 24 h in the indicated concentrations (**A**). BrdU proliferation assay: both cell lines were incubated for 24 h with different drug concentrations and were correlated with the respective control (**B**). Stars represent significance vs. nontreated (*, *p* < 0.05; **, *p* < 0.01; ***, *p* < 0.001).

Statistical analysis and graphical representation of the data was carried out using GraphPad Prism software (version 8, GraphPad Software, La Jolla, CA, USA). Statistical comparisons were performed using paired *t*-test (one treatment group is compared with the control group (untreated)) or one-way ANOVA followed by Dunnett’s multiple comparisons test (two or more treatment groups (mean of each) are compared with the mean of the control group). For the viability and proliferation assays, we have followed a curve plot based on a semilogarithmic transformation representation, followed by a nonlinear regression in the case of single drug test MTTs (Figure 1A), and we present the fitting line. For all the remaining curve plots, the statistical significances based on the Dunnett’s multiple comparisons are depicted with stars. For patients’ data, a nonparametric Kruskal–Wallis test, followed by Bonferroni post hoc test, was used for comparison among several groups for non-normal distributed variables (i.e., hormone pattern and ENSAT tumor stage). Correlation between two parameters was tested by linear regression analysis. We used nonparametric Mann–Whitney U-test for comparison between two groups, as for the tumor size, which is the only significant parameter (in Table 1). Overall survival was defined as the time from the date of initial diagnosis or primary surgery to disease-specific death or last follow-up. Survival curves were obtained with Kaplan–Meier estimates, and the differences between survival curves were assessed by the log-rank (Mantel–Cox) test. For the calculation of hazard ratios (HR), two ACC groups with negative/low or positive/high *RRM1* or *RRM2* gene expression, respectively, were considered. Cut-off was settled at median + 2 standard deviations.

All results are expressed as mean ± SEM. The statistical significance was defined as *p* < 0.05 and is denoted as stars in the graphs (* *p* < 0.05; ** *p* < 0.01; *** *p* < 0.001; **** *p* < 0.0001) in all figures, if not stated otherwise.

## 3. Results

### 3.1. Initial Drug Screening

In a first step, we performed drug screenings, including a large panel of classical chemotherapeutic agents (doxorubicin (D), etoposide (E), cisplatin (C), mitotane (M), gemcitabine (G), paclitaxel (PTX)), phytochemicals (9-cis retinoic acid (RA), isoquercitrin (I)), and molecular targeted inhibitors (erlotinib (Erl), linsitinib (L), sorafenib (SF), sunitinib (S), XAV-939 (X)) and assessed their effect on cell viability (Figure 1A) and proliferation (Figure 1B). While, e.g., E or C inhibited the cell viability of NCI-H295R cells in a highly significant (E 180 uM: 2.5%; C 160 µM: 17%) and dose-dependent manner, even at extraordinarily high drug concentrations, cell viability remained high for MUC-1 (E 180 µM: 67%; C 160 µM: 70%; both *p* < 0.001 vs. NCI-H295R). Moreover, for single treatments with D, RA, Erl, X, and I, we detected comparably low or even a complete lack of toxicity in either MUC-1 alone (for D and Erl) or both tumor models (for I). Of note, M, PTX, L, S, and SF displayed at rather high dosages as single agents overall improved toxicities.

**Figure 2:**
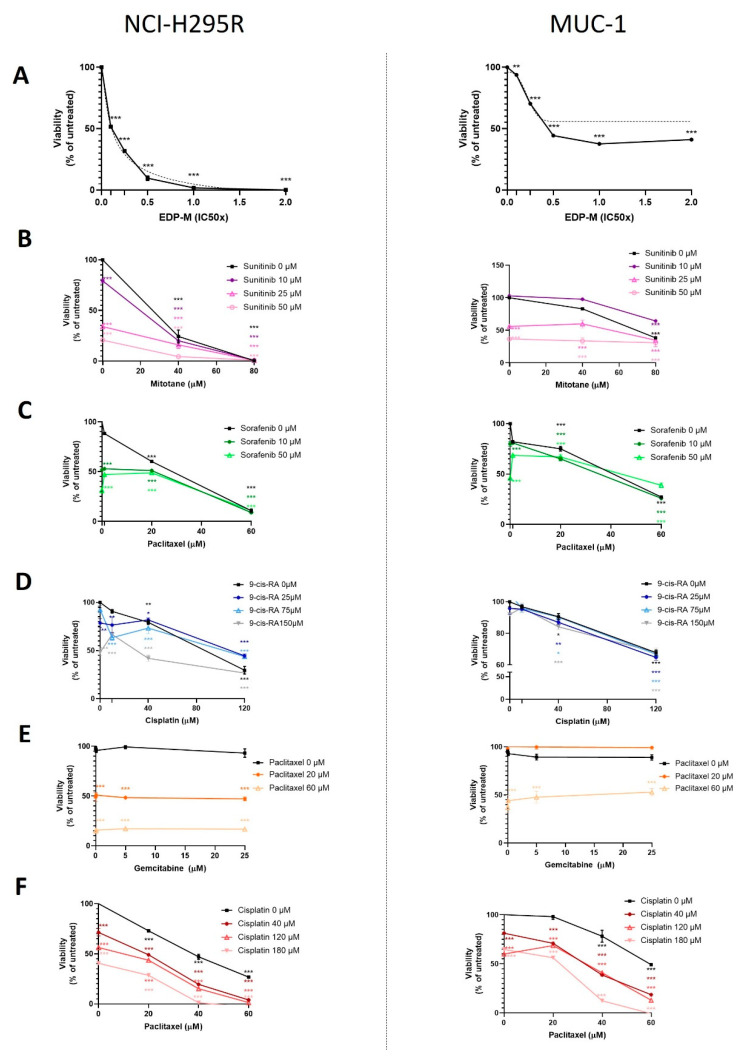
MTT viability assay for EDP-M combination in increasing concentrations for 24 h for NCI-H295R (left) and MUC-1 (right) cell lines (**A**). Specific concentrations for the separate drugs are outlined in detail in the result section. (**B**–**F**) MTT viability assays of two-drug combinations of increasing dosages vs. no drug for 24 h. Stars represent significance vs. nontreated for both treatments (*, *p* < 0.05; **, *p* < 0.01; ***, *p* < 0.001).

**Figure 3:**
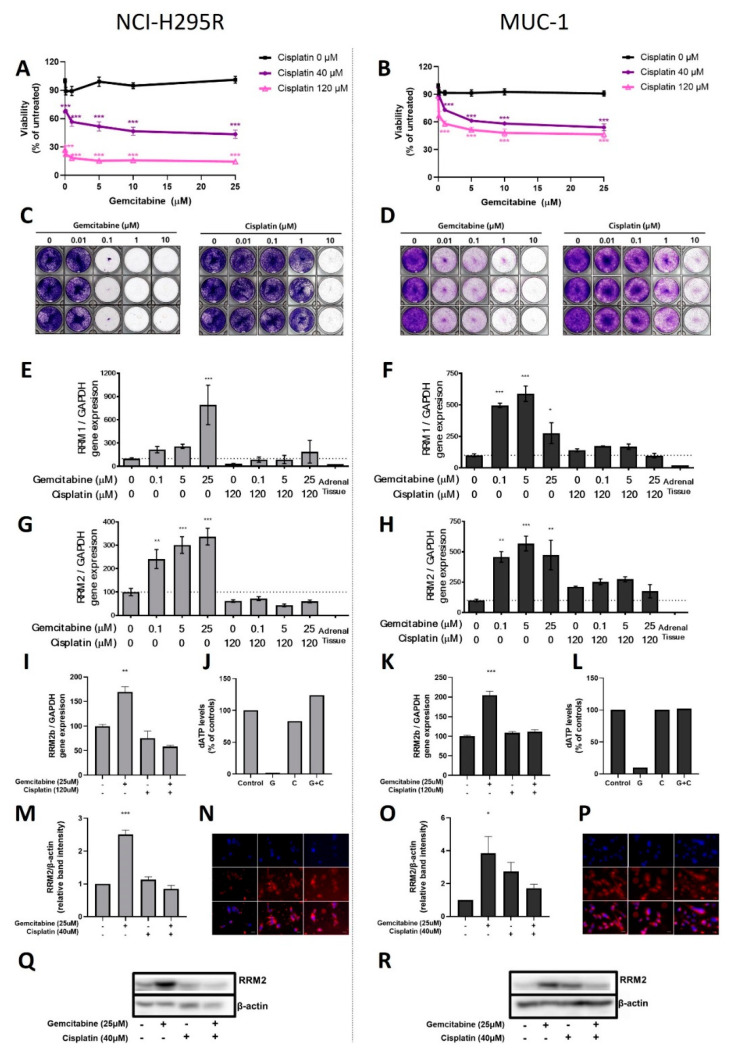
MTT viability assay for different dosages of the drug combination cisplatin and gemcitabine after 24 h of incubation for NCI-H295R (**A**) and MUC-1 (**B**) cell lines. Pictures from NCI-H295R (**C**) and MUC-1 (**D**) clonogenic assays for different concentrations of gemcitabine (left) and cisplatin (right) treatment after 24 h incubation. Real-time PCR analysis of RRM1 for NCI-H295R (**E**) and MUC-1 (**F**), as well as RRM2 (**G**,**H**) and RRM2B (**I**,**K**). dATP levels for NCI-H295R (**J**) and MUC-1 (**L**) upon different treatments. Quantification of RRM2 protein levels for NCI-H295R (**M**) and MUC-1 (**O**) and RRM2 immunofluorescence (40x) for NCI-H295R (**N**) and MUC-1 (**P**). A representative Western blot used for RRM2 protein expression quantification in NCI-H295R (**Q**) and MUC-1 (**R**) cells. Stars represent significance vs. nontreated for both treatments (*, *p* < 0.05; **, *p* < 0.01; ***, *p* < 0.001).

In a next step, we investigated appropriate combinatory treatment modalities (Figure 2). Treatment with the systemic clinical gold standard EDP-M (IC50 of EDP-M corresponds to E 60 µM, D 180 µM, C 40 µM, and M 40 µM) in a concentration of, e.g., 0.1× IC50 resulted in a highly significant decrease in NCI-H295R viability (57% *p* < 0.001), while MUC-1 displayed comparative drug resistance (95% *p* > 0.05 ns; Figure 2A). Interestingly, M and S combination led to a dose-dependent and additive reduction in cell viability in NCI-H295R, whereas in MUC-1 efficacy of single substances was partially abolished when combined (Figure 2B). While PTX as single agent was highly promising, combinations with both G or SF displayed either a lack of combinatory efficacy, or tended in MUC-1 at higher PTX concentrations of 60 µM even towards reduced efficacy compared with the single agent (Figure 2C,D). Moreover, the combination of C and RA led to rather disappointing therapeutic outcomes, either demonstrating C alone as the most effective agent or even resulting in diminished efficacies together with RA (120 µM C alone vs. combis with 25 µM and 75 µM RA, Figure 2E). From all tested combinations, two displayed promising additive antitumoral efficacy: PTX + C and G + C (Figure 2F and Figure 3A,B). At that point, simply based on the fact that generally more background knowledge was available from other cancers about the clinically commonly applied combination of G + C, potential resistance mechanisms, and druggable targets, we decided to go on, in a first step, with G + C for more detailed investigations.

In our experiments, the combination of G + C resulted in strong additive antitumoral effects for both NCI-H295R (G: 89%; C: 27%; G + C: 18%; *p* < 0.001) and MUC-1 (G: 91%; C: 88%; G + C: 58%; *p* < 0.001; Figure 3A,B). However, even though the single agents demonstrated antitumoral (Figure 1A), antiproliferative (Figure 1B) and anticlonogenic (Figure 3C,D) effects against both cell lines, and furthermore promising additive therapeutic efficacy (Figure 3A,B), remaining cell viability, and thereby drug resistance, of MUC-1 was still detectable upon combination of both drugs.

### 3.2. RRM1 and RRM2 Expression Levels in Patient Samples

**Figure 4:**
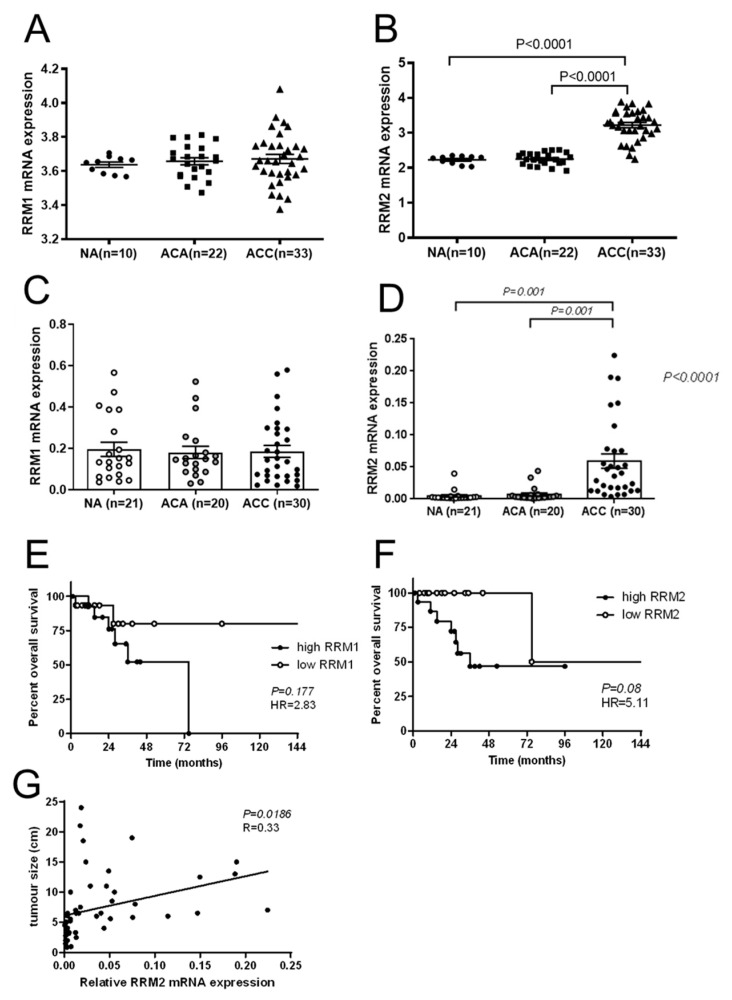
Relative *RRM1* and *RRM2* gene expression in normal adrenal (NA), adrenocortical adenoma (ACA), and adrenocortical carcinoma (ACC) samples of an available series form the literature [14] (**A**,**B**) and of our cohort (**C**,**D**). Kaplan–Meier survival curves for patients with ACC from our cohort according to low or high expression levels of *RRM1* (**E**) or *RRM2* (**F**). Correlation between the tumor size and the *RRM2* expression levels (**G**).

Based on clinically frequently observed RNR-mediated G resistance and looking for potential therapeutic molecular targets, we evaluated, in a next step, the basal expression status of the RNR subunits (RRM1 and RRM2) in ACC patient samples. Our analysis of both an available dataset (Figure 4A,B) [14] and our own patient adrenal tissue samples (Table 1, Figure 4C,D) revealed significantly elevated expression levels of *RRM2* (*p* < 0.001), but not *RRM1* gene expression in ACC (*n* = 33 and 30, respectively), compared to normal adrenals (*n* = 10 and 21) and ACA (*n* = 22 and 20). Moreover, in our cohort of adrenal tumors (ACA and ACC together), higher RRM2 expression positively correlated with larger tumor size (*p* = 0.019; R = 0.33, Figure 4G). Finally, in our ACC cohort, patients with high RRM2 expression levels at the tumor level showed a strong tendency towards poorer overall survival compared to the others (median survival 36 vs. 124 months, *p* = 0.05, HR 5.11, 95%CI 1.26–20.82; Figure 4F), while no such effect was detectable for RRM1 (Figure 4E; *p* = 0.177). Higher RRM2 expression in our patient cohort was furthermore correlated with larger tumor size (*p* = 0.019; Figure 4G).

### 3.3. RNR Subunits, RNR Activity, and Influence on Cell Viability and DNA Damage Repair

In a next step, we went on with the investigation of drug-treatment-dependent regulations of RNR subunit genes. Mechanistically, G alone induced a dose-dependent increase in expression of all relevant genes that are involved in the development of clinically relevant G resistance (RRM1, RRM2, and RRM2B; Figure 3E–I,K). Interestingly, in combination with C, these effects were significantly reversed down to basal levels or, for NCI-H295R, even below (NCI-H295R RRM1, G: 791%, *p* < 0.001; G + C: 188%; RRM2, G: 336%, *p* < 0.001; G + C: 60%, and in MUC-1 RRM1, G: 275%, *p* < 0.01; G + C: 97%; RRM2, G: 474%, *p* < 0.01; G + C: 175% vs. 100% controls). In addition, we visualized by immunofluorescence (Figure 3N,P), quantified by subsequent Western blots (Figure 3M,O), and thereby confirmed our findings, also, for regulations of RRM2 proteins (NCI-H295R, G: 2.51 ± 0.14, *p* < 0.001; C: 1.14 ± 0.85, ns; G + C 0.85 ± 0.10, ns vs. controls; MUC-1, G: 3.84 ± 0.72, *p* = 0.002; C: 2.55 ± 0.44, ns; G + C 1.57 ± 0.22, ns vs. controls; Figure 3Q,R). Moreover, we detected a treatment-dependent consumption of RNR-dependent dATP levels (NCI-H295R, G: 1.64%; C: 83.61%; G + C 124% vs. 100% of untreated controls; MUC-1, G: 13.33%; C: 101.11%; G + C 102.22% vs. 100% of untreated controls; Figure 3J,L).

**Figure 5:**
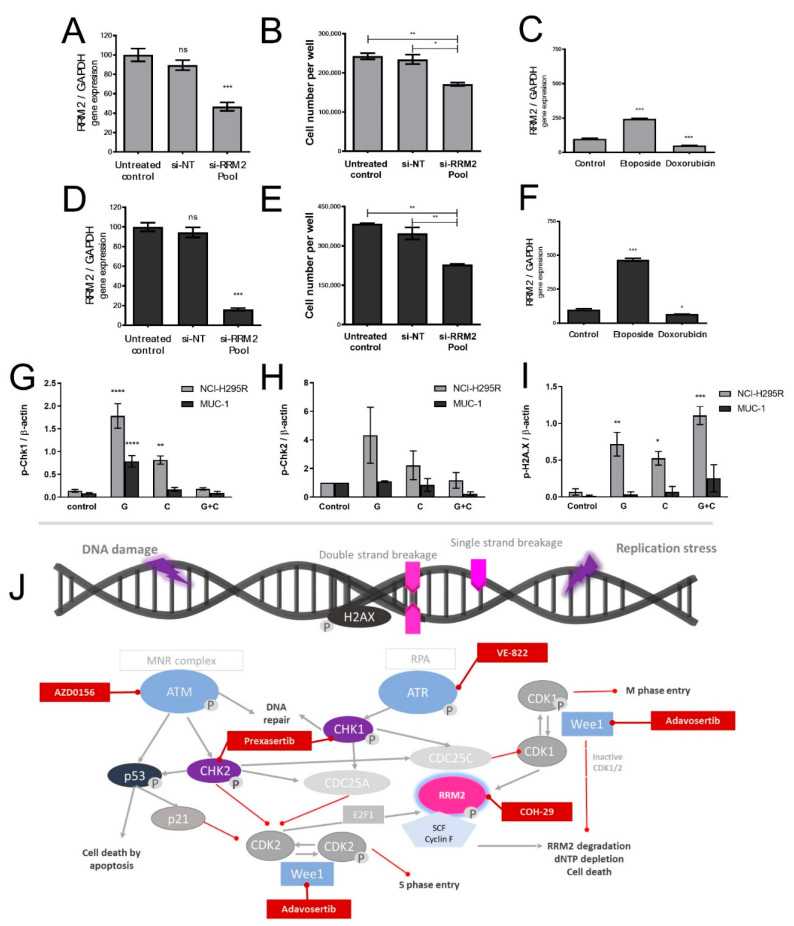
Real-time PCR analysis of *RRM2* (**A**,**D**) gene expression and number of surviving cells (**B**,**E**) under RRM2 siRNA knockdown for NCI-H295R and MUC-1, respectively. *RRM2* gene expression upon etoposide and doxorubicin treatment for NCI-H295R and MUC-1 (**C**,**F**). Quantification of Western blots for p-Chk1 (**G**), p-Chk2 (**H**), and p-H2AX (**I**) for no treatment, gemcitabine, cisplatin, and combination of both (gemcitabine and cisplatin) 24 h after treatment. Schematic illustration of the related DNA damage–repair pathway, including relevant therapeutic inhibitors (**J**). Stars represent significance vs. nontreated (*, *p* < 0.05; **, *p* < 0.01; ***, *p* < 0.001; ****, *p* < 0.0001); ns, non-significant.

**Figure 6:**
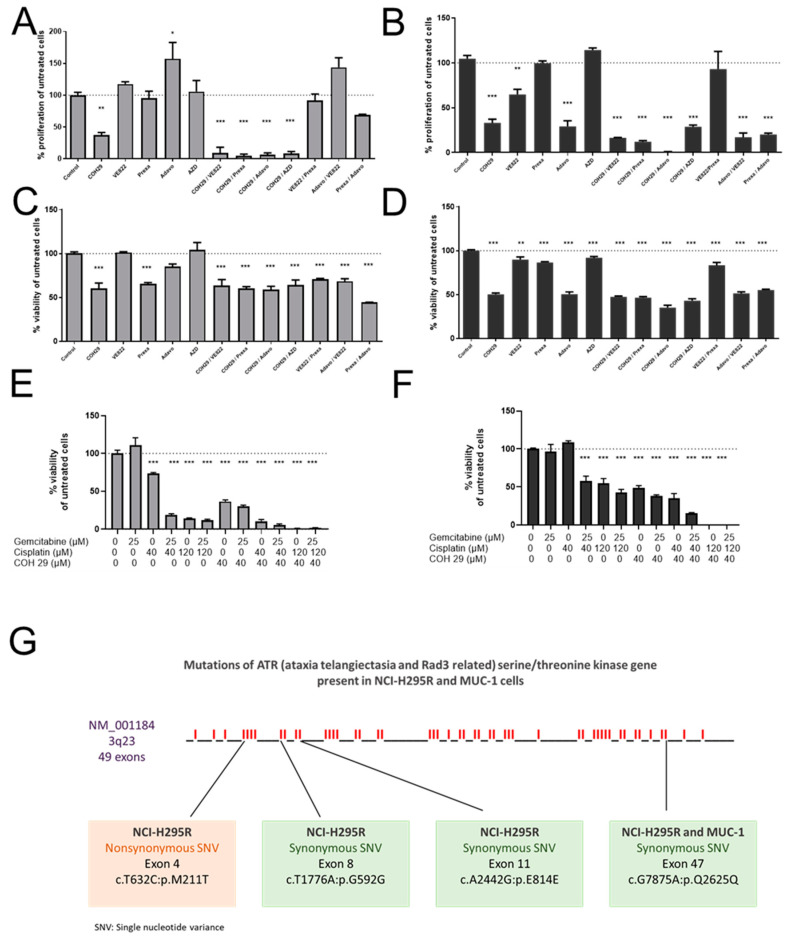
Cell viability and proliferation assays: NCI-H295R (**A**,**C**) and MUC-1 (**B**,**D**) cell lines were incubated with different inhibitors of the ATM/ATR signaling pathway. Stars represent significance vs. nontreated (*, *p* < 0.05; **, *p* < 0.01; ***, *p* < 0.001). Cell viability assays of NCI-H295R and MUC-1 cells upon treatment with different dosages of gemcitabine, cisplatin, and COH29 (**E**,**F**). Stars represent significance vs. nontreated (*, *p* < 0.05; **, *p* < 0.01; ***, *p* < 0.001). Mutations of the ATR gene detected in the NCI-H295R and MUC-1 cell lines (**G**).

In accordance with a suggested impact of RRM2 expression levels on tumor cell viability, specific siRNA-mediated RRM2 silencing (Figure 5A,D) indeed led to an accompanying significant decrease in cell count in both models (*p* < 0.001) (Figure 5B,E). Moreover, we found that not only G is able to induce specific RRM2 upregulation in both ACC models, but also etoposide (Figure 5C,F). As chemotherapeutic drugs and their related mechanisms of action are often directly associated with modulations of, e.g ATM/ATR-controlled DNA damage repair, we investigated, in a next step, relevant targets. Our investigations revealed treatment-dependent inhibitions of the activities of checkpoint kinases 1 and 2 (Chk1 and Chk2) (Figure 5G,H). Of note, conversely, the levels of p-H2AX, a sensitive molecular marker of DNA damage, increased (Figure 5I). Genetic analysis of the ATR gene in both cell lines revealed, furthermore, a nonsynonymous single nucleotide change in NCI-H295R cells (position: 142′281′612 on Chr 3:A is replaced by G, NM_001184:exon4:c.T632C:p.M211T). Additionally, two synonymous mutations have been detected in NCI-H295R cells, and one more synonymous alteration was detected in both cell lines, as depicted in Figure 6G.

### 3.4. Implementation of Complementing Molecular Inhibitors

To selectively target important candidates of the previously demonstrated highly regulated pathway components, and to furthermore overcome the remaining drug resistance of MUC-1 upon G + C treatment, we screened for complementing inhibitors of the DNA damage/repair system, directly targeting RNR, Wee1, CHK1/2, ATM, and ATR kinases, which act as schematically depicted in Figure 5J. Many of the tested inhibitors already demonstrated in MTT assays, as single agents, significant antitumor potential (NCI-H295R: untreated 100.0 ± 1.9; COH29 (40 uM, dual RRM1/RRM2-complex inhibitor): 60.2 ± 6.2%, *p* < 0.001; adavosertib (650 nM, Wee1 inhibitor): 84.9 ± 3.0%, *p* = 0.107; prexasertib (625 nM, CHK1/2 inhibitor): 65.61 ± 1.5%, *p* < 0.001; VE822 (500 nM, ATR inhibitor): 101.2 ± 0.7%, *p* = 0.999; AZD0156 (72.5 nM, ATM inhibitor): 104.3 ± 8.2%, *p* = 0.995, Figure 6A; MUC-1: untreated 100.0 ± 1.0; COH29: 50.2 ± 1.5%, *p* < 0.001; adavosertib: 50.5 ± 2.6%, *p* < 0.001; prexasertib: 86.5 ± 0.9%, *p* = 0.002; VE822: 89.7 ± 3.1%, *p* = 0.024; AZD0156: 91.9 ± 1.3%, *p* = 0.119; Figure 6B). Similar effects were observed for cell proliferation in specific BrdU assays (Figure 6C,D). However, the combination G, C, and COH29 resulted in previously unreached total cell killing of both NCI-H295R (untreated: 100.0 ± 4.3; G: 110.7 ± 20.1%, *p* = 0.196; C: 14.3 ± 0.4%, *p* < 0.001; G + C: 11.7 ± 1.2%, *p* < 0.001; G + C + COH29: 0.0 ± 0.1%, *p* < 0.001; Figure 6E) and the commonly rather highly drug-resistant MUC-1 cells (untreated: 100.0 ± 1.3; G: 96.4 ± 9.6%, *p* = 0.996; C: 57.9 ± 6.3%, *p* < 0.001; G + C: 47.2 ± 4.2%, *p* < 0.001; G + C + COH29: 0.0 ± 0.1%, *p* < 0.001; Figure 6F).

## 4. Discussion

Despite multiple studies, no effective targeted treatment is yet available for patients with advanced ACC. However, in this context, it is relevant to mention, that the preclinical screening platform in the field of ACC was inadequate for a long time, as we faced a huge lack of tumor models, with NCI-H295R as the only available cell line of human origin for more than 20 years [17]. In the current study, we have performed an extensive preclinical drug screening and subsequent mechanistical investigation based on classical chemotherapies and innovative targeted agents, using two different adrenocortical cancer cell lines: the classical NCI- H295R cell line and the more recently developed and highly drug-resistant MUC-1 cell line [11,18,19,20,21,22,23].

In a first step, the classical compounds gemcitabine and cisplatin were identified as a promising combination, since single and additive antitumoral effects could be confirmed for both models using different read-outs. Gemcitabine-based chemotherapies in combination with drugs, such as capecitabine, streptozotocin, erlotinib, and 5-FU, have been previously used in patients with progressed ACC, and they were well tolerated, but modestly to limitedly active in these combinations [24,25,26]. Of note, during these studies, RRM1 protein was, in recent years, already in the focus of interest. However, RRM1 could not be identified as a reliable prognostic marker based on the investigation of collected patient samples, which were correlated with later outcome. Of note, during these studies, RRM1 protein was in recent years already in the focus of interest. However, RRM1 could not be identified as reliable prognostic marker basing on the investigation of initially collected patient samples which were correlated with later outcome. Of particular interest in this context, such tumor samples are commonly collected by surgery before subsequent systemic therapy. Thus, if not applied in a second-line therapy, samples were also obtained before any kind of therapeutic treatment. Our findings, however, clearly indicate that there are strong modulations of RRM1, 2, and 2B gene expression, protein abundance, and subsequent activity in ACC models during/upon therapy by various chemotherapeutic drugs, such as gemcitabine, cisplatin, etoposide, and doxorubicin. Our experiments indicate, thus, that these drug-induced amplitudes might have a much higher influence than the original basal patient levels.

In accordance with our findings on strong correlations with larger tumor size and survival outcomes, RRM2 was also previously found to be well correlated with Ki67 labeling index in ACC samples, and was already considered as a potential pharmaceutical target in ACC, as *RRM2* was also found to be upregulated in NCI-H295 upon gemcitabine treatment [10]. However, in this study, the combination with other drugs, such as mitotane and 9-cis retinoic acid, did not lead to any additive therapeutic efficacy. Accordingly, mitotane and 9-cis retinoic acid demonstrated no effects on *RRM2* expression. The authors of this study concluded in 2016 that *RRM2* upregulation upon gemcitabine treatment may contribute to an emerging chemoresistance against gemcitabine, which should be overcome for successful further clinical applications. However, in this study, no potential drug candidate was found, as mitotane and 9-cis retinoic acid failed as potent partners. Our experiments confirm that some drugs, such as gemcitabine and etoposide, have the potential to upregulate *RRM2* expression, while others (cisplatin and doxorubicin) do not. RRM2 knockdown via siRNA alone resulted already in cell growth inhibition in both ACC models. Of main importance, however, is that we identified with cisplatin the potent combination partner, as it leads to additive antitumoral effect in NCI-H295 and the drug-resistant MUC-1 cells, effects which are significantly accompanied by an overall reduction in RNR subunit expression and abundance, as well as RNR and DNA damage repair activity. Of note, the general therapeutic potency of gemcitabine and cisplatin combination is not new or limited to ACC. Combination of both treatments has been tested as highly effective, and is in use for progressed cases in different organs, such as in the gallbladder, nasopharyngeal, biliary, non-small cell lung [27,28,29], among others. However, even though combined treatment is highly potent compared with gemcitabine treatment alone [29], additional treatments are often still required [28].

Thus, to potentiate the therapeutic effects of gemcitabine and cisplatin specifically on a molecular level, we focused our attention, in a next step, on the ATM/ATR pathway that contributes to the DNA damage response in cancer and upon various types of chemotherapies, including antimetabolites [30]. Indeed, we could show notable p-Chk1 and p-Chk2 increases in protein level upon G treatment, which were, in accordance with our previous findings, reversed upon C addition. In addition, we have reported a concomitant increase in p-H2AX protein upon the combinatory treatment as a marker of the excessive DNA damage. Of note, we observed that this effect was more evident in NCI-H295R than in the MUC-1 cell line. This might be explained due to various synonymous and nonsynonymous ATR mutations which were identified during our studies in NCI-H295R cells in contrast to MUC-1 cells. Cells with defective levels of ATR signaling commonly show a highly increased sensitivity to replication stress and many DNA-damaging agents and antimetabolites, such as gemcitabine [31,32]. This could explain, furthermore, the frequently observed higher drug sensitivity of NCI-H295 compared to MUC-1 [11,18,19,20,21,22,23]. However, as there is also a strong interplay between ATR and the p53 status [33], and as both cell lines carry, furthermore, different p53 mutations, this needs to be investigated in more detail in further studies. For this purpose, we are currently planning specific CRISPR/Cas mutant strains of both NCI-H295 and MUC-1.

In a next step, we performed a screening with inhibitors specifically targeting the involved ATM/ATR pathway, including also RNR. We have selected inhibitors that performed elevated levels of DNA damage on a molecular level and are currently under investigation in at least phase I clinical trials [34]. More specifically, we have tested prexasertib, an intravenously administered Chk1/Chk2 inhibitor which recently demonstrated promising clinical activity in heavily pretreated BRCA wild-type patients with recurrent ovarian cancer [35] and is currently part of multiple combinatory clinical trials. We have also included in our screening adavosertib, the only available Wee-1 inhibitor [34]. To date, adavosertib is under investigation in multiple clinical trials and has recently been shown to result, in combination with gemcitabine and radiotherapy, in increased survival rates in patients with advanced pancreatic cancer [36]. We have also tested berzosertib (VE-822), an ATR inhibitor, which recently, however, in phase I clinical trials, did not result in significant results in different tumors in combinatory treatments [37]. To inhibit ATM we have also included AZD0156 in our screening, which is currently under investigation in clinical trial (NCT02588105).

Among the different tested inhibitors of the ATM/ATR pathway, most of them demonstrated significant antitumor efficacy as single agents. However, most potent was, for both cell lines, an aromatically substituted thiazole compound (N-(4-[3,4-dihyrophenyl]-5-phenylthiazol-2-yl)-3,4-dihydroxybenzamide) and inhibitor of the dual complex of the RNR (RRM1/RRM2 complex inhibitor), named COH29 [38]. Previous studies in the epidermal carcinoma cell line and gemcitabine-resistant clones of the same line compared the activity of COH29 to the clinically established RNR inhibitors HU and to gemcitabine. It has been shown that COH29 has similar antiproliferative activity to G and is over 20-fold more active than HU [39]. Other studies highlight that COH29 can activate DNA damage checkpoints and can suppress DNA repair functions without significant genotoxicity [38]. A relevant study on prostate cancer, which had integrated data from cell lines and clinical cohorts, revealed that COH29 inhibited oncogenic activity in vitro and could be a potential therapeutic option for this type of cancer, since *RRM2* upregulation was related with poorer outcome [40]. Currently, COH29 is under investigation in a phase I trial in treating patients with solid tumors that are refractory to standard therapy, or for which no standard therapy exists (NCT02112565).

Finally, the combination of gemcitabine, cisplatin and COH29 led, in our studies, to a previously unreached total cell killing, and indicates, thereby, not only a promising strategy for ACC, but also a new strategy to potentiate therapeutic efficacy for other tumor types for which gemcitabine and cisplatin are commonly in clinical use.

Overall, our results begin to shed new light on the involvement of RNR activity in the response to ACC therapy and propose that RNR-modulating therapies might represent a new therapeutic option for ACC and other cancers.

## 5. Conclusions

Although further in vivo studies are needed, our findings indicate that a combination of gemcitabine, cisplatin, and molecular inhibitors, such as COH-29, targeting the ATM/ATR/RRM axis might have the potential for a next level of chemotherapeutic treatments in ACC. By detailed molecular characterization of the underlying drug resistance mechanisms of gemcitabine treatment and the pathway-specific addition of complementing cisplatin, but also molecular inhibitors, our experiments provide evidence that RNR-modulating therapies might represent a new therapeutic option for ACC.

## Figures and Tables

**Table 1:** Demographic data and clinical parameters for patients with adrenocortical tumors (*n* = 50).

Variable	ACA (*n* = 20)	ACC (*n* = 30)	*p*
Sex (m/f)	7/13	14/16	NS
Age (years, median)	50	51	NS
Tumor size (cm, median)	3.3	7.5	<0.001
**Hormone Pattern (*n*)**			NS
Mixed	0	3	
Cortisol excess	12	11	
Aldosterone excess	2	0	
Androgen excess	0	0	
Inactive	6	13	
Unknown	0	3	
**ENSAT Tumor Stage (*n*)**			
1–2	NA	12	NA
3		10	
4		6	
Unknown		2	
Weiss score (median)	NA	7	NA
Ki-67 (%, median)	NA	20	NA

ACA, adrenocortical adenoma; ACC, adrenocortical carcinoma; m, male; f, female; NS, non-significant; n, number; NA, not applicable; tumor stage according to the European Network for the Study of Adrenal Tumors (ENSAT) classification [1,15].

## Data Availability

Not applicable.

## Supplementary Materials

The following are available online at https://www.mdpi.com/article/10.3390/cancers13164200/s1, Figure S1: Western Blots used for the pictures in Figure 3Q,R as well as for the graph genera-tion in Figure 3M (NCI-H295R) and Figure 3O (MUC-1)., Figure S2. Western Blots used for the graph generation in Figure 5G, Figure S3. Western Blots used for the graph generation in Figure 5H, Figure S4. Western Blots used for the graph generation in Figure 5I.
