# Supplementary material for: Novel Insights into the Molecular Regulation of Ribonucleotide Reductase in Adrenocortical Carcinoma Treatment
Source: Cancers (Basel). 2021 Aug 20;13(16):4200. doi: 10.3390/cancers13164200 (PMC8391410; doi:10.3390/cancers13164200)
Supplement: Supplementary file 1 [file cancers-13-04200-s001.zip › cancers-1271302-supplementary.pdf]

cancers

MDPI

Supplementary Material: Novel Insights into the Molecular Regulation of Ribonucleotide Reductase in Adrenocortical Carcinoma Treatment

Christina Bothou, Ashish Sharma, Adrian Oo, Baek Kim, Pal Perge, Peter Igaz, Cristina L. Ronchi, Igor Shapiro and Constanze Hantel

A. RRM2 (results provided normalized for untreated band intensity)

RRM2

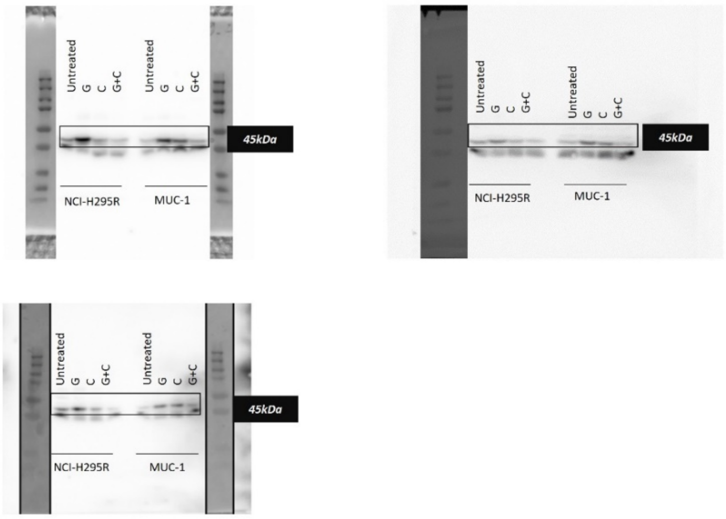

β-actin

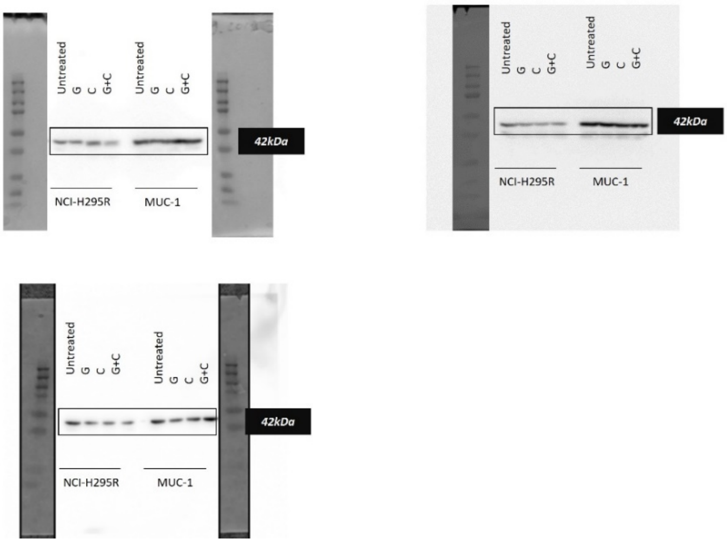

Figure S1. Western Blots used for the pictures in Fig.3Q and 3R as well as for the graph generation in Fig.3M (NCI-H295R) and 3O (MUC-1).

B. p-Chk1

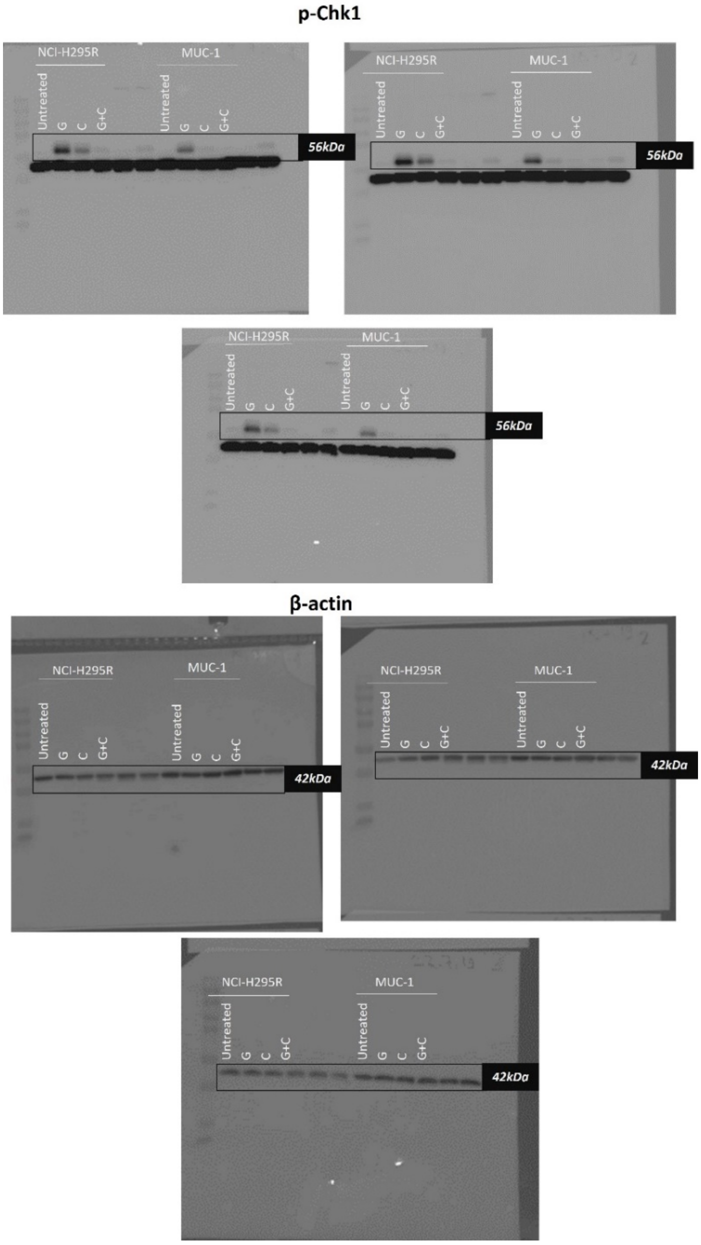

Figure S2. Western Blots used for the graph generation in Fig.5G.

C. p-Chk2 (results provided normalized for untreated band intensity)

p-Chk2

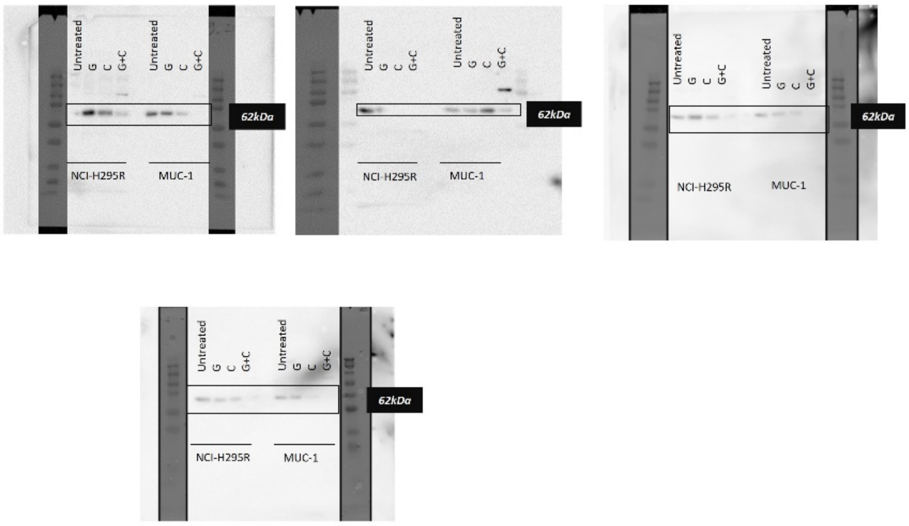

β-actin

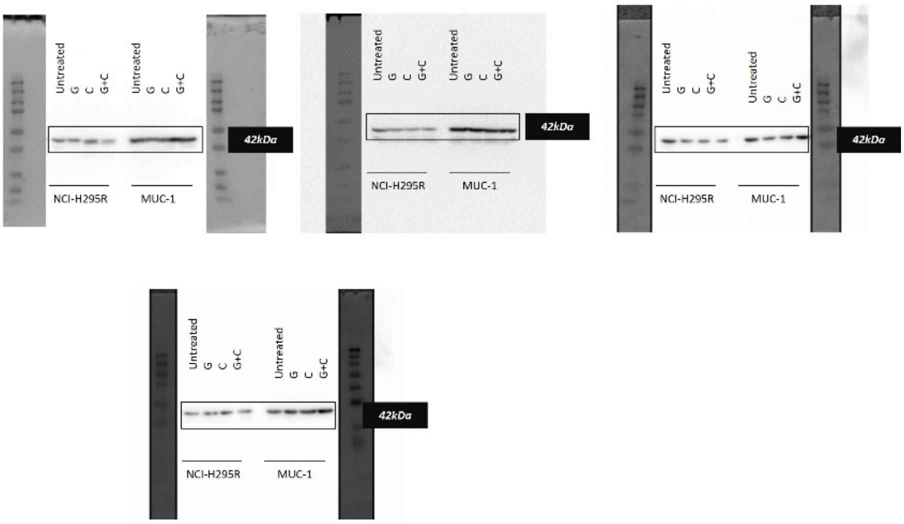

Figure S3. Western Blots used for the graph generation in Fig.5H.

D. p-H2A.X

p-H2A.X

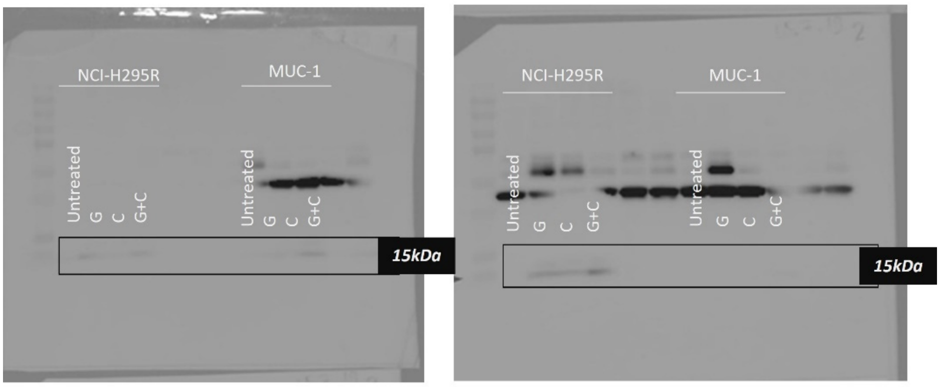

β-actin

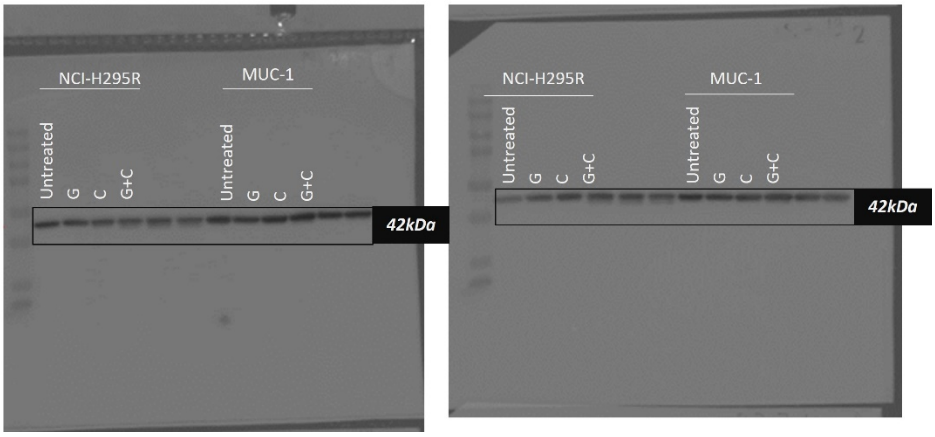

Figure S4. Western Blots used for the graph generation in Fig.5I.

For all Blots:

- G: Gemcitabine 25 μM; C: Cisplatin 40 μM; G+C: Gemcitabine (25 μM) and Cisplatin (40 μM).
- For molecular weight estimation Precision Plus Protein Dual Color Standards (#1610374, Bio-Rad) has been used.
